# Encouraging Healthier Food and Beverage Purchasing and Consumption: A Review of Interventions within Grocery Retail Settings

**DOI:** 10.3390/ijerph192316107

**Published:** 2022-12-01

**Authors:** Henry Wolgast, McKenna M. Halverson, Nicole Kennedy, Isabel Gallard, Allison Karpyn

**Affiliations:** 1 Center for Research in Education and Social Policy, University of Delaware, Newark, DE 19716, USA; 2 Temple University, Philadelphia, PA 19122, USA

**Keywords:** dietary behaviors, dietary intake, food access, healthier food, nutrition, retail food environment, review

## Abstract

This review identifies the most promising intervention strategies for promoting the purchase and consumption of healthier items within U.S. grocery retail settings, with a particular focus on those strategies that may be most effective when implemented within SNAP-authorized retail settings. Searches of nine electronic databases, as well as forward and backward searches, yielded 1942 studies. After being screened, 73 peer-reviewed academic articles were identified for inclusion. Of these, 33 analyzed single-component interventions, while 40 assessed multi-component interventions. The following unique intervention types were considered as evaluated in these studies for their ability to increase healthy item purchasing and consumption: (1) nutrition scoring, (2) nutritional messaging, (3) non-nutritional messaging, (4) endcaps and secondary placement, (5) point-of-sale interventions, (6) increased stocking, (7) food tasting and demonstrations, (8) nutrition education, and (9) placement on shelf interventions. Nutritional scoring and nutritional messaging emerged as the most rigorously tested and effective intervention strategies. Other strategies warrant more research attention. Simple intervention strategies, as opposed to complex ones, yield the most successful results and minimize shopper burden. Therefore, these strategies should be reviewed for policy implementation within SNAP-authorized grocery retailers.

## 1. Introduction

As concerns for global dietary quality persist, public health experts continue to evaluate the role that individuals’ environments play in shaping their health behaviors [1,2,3]. Internationally, concerns for malnutrition in all its forms, has brought attention to the important connections between obesity and undernutrition on health. Preventing poor dietary quality is a leading priority for a wide variety of chronic diseases, and a critical disease mitigation strategy globally. Food retail is an important nexus between food policy and the consumer and as such has remained a critical facet of food environment intervention [4,5].

Grocery retail stores are one aspect of individuals’ environments that have the potential to directly impact their dietary quality [6,7]. Research demonstrates that these stores vary widely in their availability of, and strategies used to promote, healthy options [8,9,10]. Findings from individual studies demonstrate that manipulating certain aspects of these environments (e.g., placing healthy items at eye level, installing freezers to increase a store’s stock of fresh fruits and vegetables) can promote healthier purchasing behaviors and consumption patterns among shoppers [11]. Multiple studies have been conducted across a range of countries which examine how supermarket environments influence promotion and purchasing of less healthy food, however fewer examine the promotion of healthier items [12,13].

In the United States, there is potential to impact the retail environment at the federal level through the Supplemental Nutrition Assistance Program (SNAP), in which over 250,000 grocery retailers participate across the United States. SNAP-authorized grocery retailers profit substantially from the program, with $61 billion in SNAP sales representing approximately 8 percent of grocery sales industry-wide [14]. Despite evidence demonstrating that interventions within grocery retail settings can improve the healthfulness of shoppers’ purchases and consumption, the U.S. Department of Agriculture (USDA), which has the authority to authorize SNAP grocery retailers and establish eligibility criteria, requires little from grocery retailers to participate beyond stocking minimal staple foods.

At the same time, disparities in dietary quality by income persist. For example, research demonstrates that disparities in the price of nutrient-dense foods versus less nutrient-dense foods are rising [15]. Specifically, studies show that ultra-processed foods tend to be less expensive, though more energy dense, than unprocessed foods [16]. These cost barriers contribute to socioeconomic inequities in dietary quality, leading individuals with lower incomes to have less choice in food purchasing, and worse dietary outcomes [15,17].

The purpose of the present study is to provide an updated review of healthy food marketing strategies in retail stores, with a focus on research conducted in stores accepting SNAP benefits [18]. Further, the study goes beyond prior reviews, which broadly characterize the effectiveness of interventions according to the 4 P’s of marketing, to examine specific strategies and their effect on consumer purchasing, consumption or sales. Such information is intended to guide future research and interventions in retail settings.

## 2. Methods

This review used the Preferred Reporting Items for Systematic Reviews and Meta-analyses (PRISMA) guidelines.

### 2.1. Search Strategy

The authors used several methods to ensure a thorough and comprehensive review of the literature on in-store marketing interventions for healthy food and beverage promotion. First, a list of inclusion criteria was created to identify papers to be included in the review sample. Second, a list of key terms was created to search for studies. Third, appropriate databases were identified for the search based on the database topics. Finally, a database search was conducted to identify inclusion articles, using both forward and backward searches for each inclusion article. Below are the processes used to identify studies for this review.

### 2.2. Inclusion Criteria

The studies included are original empirical research published between 1 January 2010 and 6 June 2022, in English, and from the United States. The authors chose to focus on the United States for this review because SNAP-authorized retail contexts are unique to this country. Studies were researcher- or retailor-initiated, conducted inside the physical grocery retail environment, and manipulated the grocery retail environment. Evaluations could be quantitative or use mixed methods. All interventions had to include at least one of the following outcomes: (1) purchase-related (i.e., objective store sales data, objective food purchasing data, customer receipts, and survey self-reported purchases or expenditures, store sales, or intent to purchase), and/or (2) consumption-related (i.e., food frequency questionnaires (FFQ), 24 h dietary recalls, food diary, Veggie Meter^TM^ or other biometrics, or other self-reported diet/consumption or intent to eat surveys).

### 2.3. Exclusion Criteria

Interventions were excluded if they were implemented by an entity other than a researcher or grocery retailer (e.g., price intervention at the wholesale level or front-of-pack labels initiated by a food company), if they did not occur inside the physical grocery retail environment (e.g., restaurants, schools, mobile food trucks, online, and laboratory), or if they did not manipulate the grocery retail environment (e.g., grocery store tours). Additionally, articles were excluded that solely utilized price-based interventions, namely those implementing a coupon or discount-based behavioral nudges.

### 2.4. Search Terms and Databases

Nine databases (i.e., Academic OneFile, Business Source Premier, CAB Abstracts, Communication and Mass Media Complete, Family and Society Studies Worldwide, PsycINFO, PubMed, Sociological Abstracts, and Web of Science) from a variety of sectors (i.e., agriculture, business, communication, health, and psychology) were searched. Key terms were constructed based on three concepts: (1) healthier food, (2) study design, and (3) setting. A variety of search terms were used to ensure articles would be included with nuanced differences in terms (e.g., healthy food vs. better-for-you) across sectors. The following key terms were used in all database searches:
Healthier food“health* food*” OR “healthy eating” OR “fruit*” OR “vegetable*” OR “low* fat” OR “low* sodium” OR “low* sugar” OR “low-fat” OR “low-sodium” OR “low-sugar” OR “better for you” OR “nutritio*”Study design“intervention” OR “pilot” OR “experiment*”Setting“supermarket*” OR “grocery store*” OR “corner store*” OR “bodega*” OR “retail environment”

### 2.5. Procedure of Article Search

**Figure 1:**
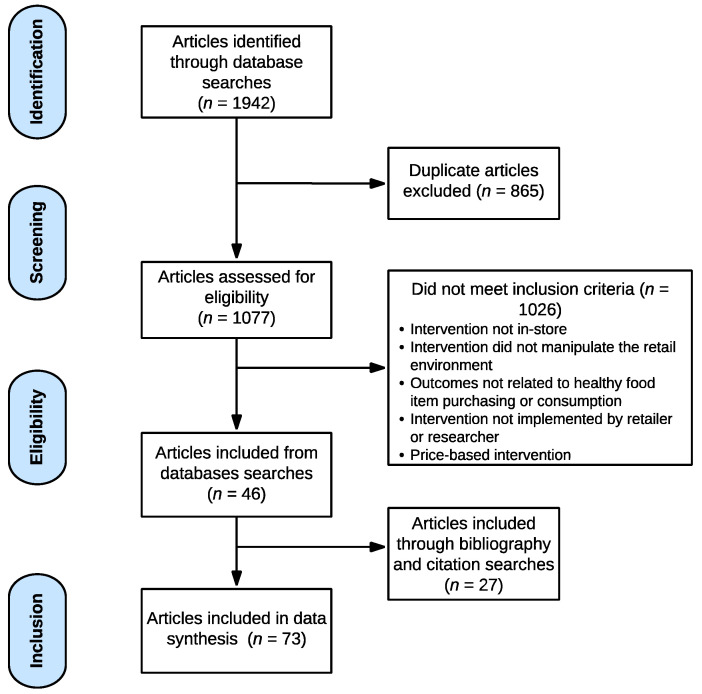
Article Inclusion Process.

The RefWorks database was used to organize all articles [19,20]. The searches were conducted by two authors and yielded 1942 studies (Figure 1). After excluding 865 duplicate articles, five coders reviewed each full-text article to determine eligibility and excluded 1026 studies. This review yielded 46 articles that met all inclusion criteria. Then, citation and bibliography searches were conducted with all 46 articles identifying an additional 27 articles for a final total of 73 articles. Of all the article articles reviewed, 38 journals were represented. The journals represented most frequently were Journal of Nutrition education and Behavior, Public Health Nutrition, and Preventive Medicine Reports.

After removing duplicates, five reviewers independently screened the title, abstract, and full text of the remaining 1077 articles. Reviewers discussed any differences and consulted a sixth reviewer, when necessary, and a consensus was reached. Five reviewers conducted forward and backward searches of the included articles. Titles and then full texts were reviewed to assess eligibility. Articles were abstracted and coded independently with five coders; discrepancies were discussed until a consensus was reached. Article abstractions included participants, study design, intervention description, intervention type, intervention setting, duration of intervention, data collection methods, outcome variables, and key findings.

Researchers reviewed the studies and categorized them by intervention type. The following unique intervention types were evaluated for their ability to increase healthy item purchasing and consumption: (1) nutrition scoring, (2) nutritional messaging, (3) non-nutritional messaging, (4) endcaps and secondary placement, (5) point-of-sale interventions (which includes healthy checkout interventions), (6) increased stocking, (7) food tasting and demonstrations, (8) nutrition education, and (9) placement on shelf interventions (Table 1). Articles were then coded either as single-component, meaning that they evaluated the effects of one intervention strategy in isolation, or multi-component, meaning that they evaluated the effects of two or more intervention strategies alongside each other.

Articles were also coded to identify whether the intervention was conducted within SNAP-authorized retail settings. SNAP acceptance was coded based on the following categorizations: (1) the study explicitly stated that the retailer was SNAP-authorized, (2) the study was highly likely to have been conducted in a SNAP-authorized retail setting (e.g., low-income neighborhoods, corner stores, bodegas, major supermarkets), and (3) the study was not likely to have been conducted in a SNAP-authorized retail setting (e.g., high-income neighborhood) or lacked sufficient information to make a determination.

Additionally, articles were coded to identify whether they were experimental, quasi-experimental, or pre-experimental. Articles were coded as experimental if participants were randomized to conditions, quasi-experimental if participants were not randomized to conditions but there was a comparison group, or pre-experimental if participants were not randomized to conditions and there was no comparison group.

## 3. Results

There were 73 peer-reviewed academic articles identified for inclusion in this review, each of which evaluated the healthy food and/or beverage purchasing or consumption-related effects of retail interventions. These studies employed nine unique intervention types (Table 1). In total, 33 studies analyzed single-component interventions, while 40 studies assessed multi-component interventions.

### 3.1. Single-Component Interventions

#### 3.1.1. Nutrition Scoring

Eleven studies evaluated nutrition scoring (Table 2) [21,22,23,24,25,26,27,28,29,30,31]. The results of nutrition scoring interventions are largely positive. In 9 of 11 studies, at least one finding showed a significant positive effect on healthy purchasing or consumption. Among available research, findings suggest that simpler scoring systems are more effective than those with multiple nutrition facts. For example, the sole study of nutrition scoring using an experimental approach, conducted by Kiesel and Villas-Boas, found that no trans-fat labels significantly increased sales of treated products by 23 percent. Yet, labels with multiple measures of healthfulness were found to have a null effect on healthy purchasing, likely overwhelming shoppers with high information costs [26]. Five quasi-experimental studies echoed the experimental study’s increased effectiveness in simpler nutritional scoring systems, including studies of the NuVal and Guiding Stars program which both assign a single value to the healthfulness of a product [21,22,23,28,29]. For example, Rahkovsky et al. found that the Guiding Stars Program decreased sales of unstarred (least healthy) products at intervention stores by 2.58 percent, while also increasing sales of 1-star, 2-star, and 3-star cereals by 1.15 percent, 0.89 percent, and 0.54 percent. Five studies of nutrition scoring were pre-experimental [22,24,25,27,30]. Additionally, 10 studies collected objective data [21,22,24,25,26,27,28,29,30,31]. Of the eleven interventions that utilized a nutrition scoring component, ten were conducted in a retail setting that explicitly or likely accepts SNAP benefits [21,22,23,24,26,27,28,29,30,31].

#### 3.1.2. Increased Stocking

Six studies evaluated single-component interventions in which stores increased their stock of healthy foods and beverages (Table 2) [32,33,34,35,36,37]. None of the studies evaluating increased stocking interventions were experimental. Less rigorous studies using quasi-experimental and pre-experimental designs demonstrate that, although increased stocking interventions tend to result in an increased supply of healthy foods and beverages offered in stores, the interventions generally do not have a significant impact on participants’ healthy food and beverage purchasing or consumption [35,36,37]. For example, the North Carolina Healthy Food Small Retailer Program (HFSRP) is an intervention in which small food stores were provided with up to $25,000 to spend on refrigeration units to increase their stocking of healthy foods and beverages (e.g., fresh fruits and vegetables, lean meats, whole grains, etc.) [32,33,35,36]. Results of quasi-experimental studies evaluating this intervention demonstrate that after one year of implementation, Healthy Food Supply (HFS) scores, a measure of availability of healthy items within a store, increased by 3.13 points between baseline and follow-up among HFSRP stores, whereas HFS scores decreased by 0.44 points in control stores. However, participants’ purchases, skin carotenoids, and self-reported consumption did not significantly differ between HFSRP stores and control stores. Additionally, 4 of 6 articles assessing increased stocking interventions collected objective purchasing data. Of the studies evaluating increased stocking interventions, four were conducted in stores that explicitly served SNAP customers [32,33,36,37], and one was located in a low-income community that likely served SNAP customers [34].

#### 3.1.3. Nutritional Messaging

Five studies evaluated the effect of nutritional messaging techniques (Table 2) [38,39,40,41,42]. Two studies employed experimental designs [40,42]. Two studies were quasi-experimental [38,41], and one study was pre-experimental [39]. Four nutritional messaging studies measured outcomes using objective data [38,40,41,42]. Of the two experimental studies examining purchasing and sales data, both found mixed effects including a significant increase in healthy item purchasing despite no change in healthy item consumption [40,42]. Further, results were largely dependent on the food group under study [40]. Results from quasi- and pre-experimental studies, however, were predominantly positive. For example, Finnell et al. conducted a marketing campaign, 1% Low-Fat Milk Has Perks!*,* that included dairy case clings, souvenir buttons, and a handout with nutrition information entitled “Lactaid Factoids.” They found that the campaign significantly increased lower fat milk sales at intervention stores 4.8 percent over baseline [38]. Moreover, three of the five studies examining nutritional messaging were explicitly [42] or highly likely [41] to have been conducted in a retail setting that accepts SNAP benefits [40].

#### 3.1.4. Non-Nutritional Messaging

Four studies evaluated the effect of non-nutritional messaging techniques yielding mixed results (Table 2) [43,44,45,46]. Of the two studies that employed experimental designs, one found positive effects on healthy food purchasing while the other yielded mixed effects [44]. Results of two quasi-experimental studies found positive and negative results [45,46]. In the most successful non-nutritional messaging intervention, Payne et al. applied behavioral economics principles and created placards for grocery carts that listed both the average number of produce items purchased at the store as well as the top ten fruits and vegetables purchased in that store to “give shoppers a specific idea of not only the appropriate or normal amount of fruits and vegetables to purchase, but also the most common types of fruits and vegetables purchased” [46]. Findings reported an increase in shopper spending on produce between 7.5 and 16 percent. However, Chapman et al. found non-nutritional messaging labels and floor stickers unsuccessful at increasing healthy purchasing [45]. Specifically, Chapman et al. found that both scarcity labeling of healthy items as well as floor labeling guiding shoppers to healthier store sections led to no significant increase in healthy food sales. One additional non-nutritional messaging approach, which included the piping of the smell of cookies through a grocery store generated counter-intuitive results, whereby consumers smelling the cookies purchased a significantly higher proportion of healthier items [44]. Of the four studies examining the effects of non-nutritional messaging, all four were explicitly [45,46] or highly likely [43,44] to have been conducted in a retail setting that accepts SNAP benefits.

#### 3.1.5. Food Tasting and Demonstrations

Two studies evaluated interventions that were solely composed of food tasting and demonstrations (Table 2) [47,48]. Of these, one study was experimental [47] and one was pre-experimental [48]. Both studies reported positive effects on healthy food purchasing or consumption. The most rigorous study, which examined the effects of food tasting using an experimental design and objective data, posed the question “Can healthy samples given at a grocery store prompt healthier choices?” To answer this question, participants were divided into three groups: those that received an apple sample, those that received a cookie sample, and those that received no sample. It was found that participants who received an apple sample purchased a greater amount of fruits and vegetables (M = 2.78, SD = 2.15) than did participants who received a cookie sample (M = 2.17, SD = 2.26) or no sample (M = 2.22, SD = 2.15), providing support for the hypothesis that sampling a product that is considered healthy will increase the healthiness of purchases. Of the two articles that analyzed food tasting and demonstrations in the single-component intervention category, one article was explicitly conducted in a retail setting that accepts SNAP benefits [48]

#### 3.1.6. Nutrition Education

Two studies examined the effectiveness of single-component nutrition education intervention strategies, both of which were pre-experimental (Table 2) [49,50]. Findings were both positive and null, with both studies examining the effects of nutrition education delivered via podcasts. As a result of this intervention, there was a “clear increase” in the purchase of foods emphasized in the podcasts, which 59 percent of participants (*n* = 102) purchased more of. Furthermore, 38 percent of participants (*n* = 66) had purchased none of the emphasized foods during the 6 months prior to the intervention but did so afterward. Notably, there was no significant relationship found between intervention-day responses regarding intent to purchase the emphasized foods and actual purchases [49]. A smaller pilot study conducted by the same authors corroborated the conclusion that nutrition education was effective at influencing actual and planned purchasing behavior [50]. Note that while the larger study reported objective store sales data, the smaller pilot study only included subjective self-report data. In conclusion, while findings are largely positive and tested in retail settings accepting SNAP benefits, the limited number of studies demonstrates that there is limited evidence of the overall applicability of educational interventions to improve healthy purchasing and consumption.

#### 3.1.7. Endcaps and Secondary Placement

Two studies examined the effectiveness of endcaps and secondary placement on increasing healthier purchasing and consumption in retail environments (Table 2) [51,52]. One of these studies was experimental [51] and one was quasi-experimental [52]; both yielded positive outcomes. Liu et al. measured the influence of endcaps on purchasing of indulgent, healthy and neutral foods in 12 different convenience stores for which SNAP redemption is unspecified [51]. Featuring healthy products alone on the endcap increased healthy product sales by one-third. In the other study which evaluated a similar strategy, Payne & Niculescu found that SNAP participants purchased significantly more healthy items when they were displayed on endcaps. However, total produce spending in both the general population and SNAP recipients did not significantly increase [52].

#### 3.1.8. Point-of-Sale Interventions

One study implemented a point-of-sale intervention, which occurred directly in a checkout line (Table 2) [53]. Adjoian et al., using objective purchasing data, found that healthy checkout lanes are an effective method for increasing healthy purchases. For example, SNAP participants bought more than twice the amount of healthy food items (e.g., nuts, fresh produce, granola bars, etc.) in the intervention checkout lane as opposed to the control checkout lane [53].

#### 3.1.9. Placement on Shelf

There were no single-component studies examining placement on shelf interventions (Table 2). However, 13 studies evaluated placement on shelf interventions in tandem with other intervention strategies (e.g., endcaps and secondary placement, increased stocking, etc.), and are described below in the multi-component section.

### 3.2. Multi-Component Intervention

A total of 40 studies analyzed the effects of multi-component interventions on healthy food item purchasing and consumption (Table 3) [32,35,39,54,55,56,57,58,59,60,61,62,63,64,65,66,67,68,69,70,71,72,73,74,75,76,77,78,79,80,81,82,83,84,85,86,87,88]. Of these, 16 studies were experimental [55,59,60,61,62,63,64,72,73,74,82,83,86,88,89,90], 11 were quasi-experimental [35,56,57,58,65,66,67,75,76,77,84], and 13 were pre-experimental [32,39,54,68,69,70,71,78,79,80,81,85,87]. A total of 17 multi-component studies collected objective outcomes data [32,35,39,55,56,61,62,67,68,69,72,73,75,78,82,84,89]. Additionally, of the studies evaluating multi-component interventions, 14 were conducted in stores that explicitly served SNAP customers [32,54,58,60,64,67,68,72,73,78,82,85,86,89], 15 were located in a low-income community that likely served SNAP customers [39,55,56,57,62,63,69,74,75,76,77,80,83,87,90], 5 were conducted in a corner store or bodega without any neighborhood demographics or income levels disclosed [35,61,66,71,88] and 3 were conducted in major supermarkets [59,79,81]. Results of multi-component intervention studies are presented below, with the most rigorous studies (those with experimental and objective data) presented first (Table A1).

Analysis of the 7 experimental studies of multi-component interventions using objective data generally showed consistency in outcomes when compared to the single component study results. For example, multi-component studies which utilized nutrition scoring and nutrition education were also successful in a multi-component intervention. For example, Milliron et al. conducted a randomized control trial evaluating a multi-component intervention using objective sales data and found that pairing a brief front-of-store nutrition education effort with the EatSmart nutritional scoring system, which marked healthy products across the store, positively influenced the number of servings of fruit and vegetables purchased by shoppers. Specifically, this multi-component intervention resulted in shoppers purchasing 9.5 more servings of whole fruit and 4.8 more servings of dark green/bright yellow vegetables than the control group [55].

In contrast to Milliron et al., who evaluated a multi-component intervention including just two strategies, Foster et al. conducted a randomized controlled trial evaluating a multi-component intervention comprising five intervention strategies: placement on shelf, endcaps and secondary placement, food tasting and demonstrations, nutritional marketing, and increased stocking. Placement on shelf was the dominant strategy within this intervention, whereas other strategies (e.g., food tasting and demonstrations) were only applied to one of the five targeted food and beverage categories. Store sales data demonstrate that sales of skim and 1% milk, water (in aisle and at checkout), and two out of three types of frozen meals significantly increased compared to control store sales. Null results were found for the other categories (i.e., cereal, whole or 2% milk, beverages, or diet beverages). The authors concluded that simple, straightforward placement and product availability strategies were effective at significantly influencing the purchase of healthier items in several food and beverage categories [82].

In line with findings from Foster et al., several other studies show that simple placement strategies proved effective over more complex strategies. In an experimental study with objective outcome data, Williams et al. employed a wide variety of intervention techniques to encourage healthy purchasing, of which the most promising results stemmed from a low-cost placement technique. Williams et al. analyzed a multi-component intervention that combined nutritional marketing, increased stocking, and endcaps and secondary placement and found that shoppers on average doubled their weekly spending on the fruit and vegetable basket from $2.00–$3.00 to $5.00–$6.00 as a result [61].

While most multi-component interventions manipulate just one level of the food system (e.g., corner stores), researchers in Baltimore, MD manipulated several levels of the food system (e.g., wholesalers, corner stores, carry-outs, recreation centers, households) simultaneously in a community-wide effort to reduce obesity and generally found positive impacts on product availability but no effect on purchasing. Within this multi-level, multi-component intervention, called B’more Healthy Communities for Kids (BHCK), wholesalers, corner stores, and carry-outs were all asked to increase their stock of healthier products, which included low-fat, low-sugar snacks and beverages, fruits and vegetables, and whole grain products. Additionally, nutritional messaging techniques (e.g., posters and signage) were then used within corner stores and carry-outs to promote these items. Corner stores also implemented nutrition education and food tasting and demonstration strategies, which were led by BHCK interventionists and allowed customers to sample healthy food items. Intervention efforts at the wholesale level had a positive effect on sales of healthier items for resale. Intervention stores had larger increases in their stock of healthy items, as measured by the Healthy Food Availability Index, than did control scores (e.g., 5.65-point increase vs. 1.67-point increase). The intervention had no effect on adult caregivers’ healthy food purchasing [62].

Although increased stocking did not lead to increases in healthy purchasing when paired with nutritional messaging, nutrition education, and food tasting and demonstrations in the BHCK intervention [62], findings from Thorndike et al. show that efforts to improve available stock of healthy items can be successful when paired with other intervention strategies. In this randomized controlled study, three stores were randomly assigned to receive the multi-component intervention consisting of increased stocking, non-nutritional marketing, and placement on shelf, and three were randomly assigned to the control group. All stores were located in low-income communities and accepted SNAP and WIC. Each intervention store had a combination of interventions: A consultant that helped advise fresh fruit and vegetable maintenance, different supplies and advice such as display tips and items that would make fruits and vegetables more attractive to customers, and a service job provided to the stores such as refrigeration installation, or having the walls repainted. Findings showed an increase in fruit and vegetable sales by $40.00 per month by participants using WIC fruit/vegetable cash-value vouchers, whereas fruit and vegetable sales declined in the control group by $23.00 per month [73].

## 4. Discussion

Interventions to improve the healthfulness of individuals’ food and beverage purchasing and consumption within retail environments are varied and heterogeneous. Results of this review demonstrate that nutritional scoring and nutritional messaging are the most widely-researched and effective single-component healthy retail intervention strategies [24,29]. Furthermore, nutritional messaging interventions, including simple signage at the front of the store, corresponding with shelf tags for reinforcement, are generally effective [41]. Notably, both of these intervention strategies were commonly implemented within SNAP-authorized retail settings.

Two additional intervention strategies, food tasting and demonstrations and nutrition education, also demonstrate positive effects on healthy food and beverage purchasing and consumption, however, the literature in this area is sparse. Non-nutritional marketing is another intervention strategy commonly implemented within grocery retail settings, however, results from studies examining this strategy are mixed. Collectively there is emerging evidence for the use of interventions focused on endcaps and secondary placement to increase healthy purchasing and consumption, although only a small number of articles (*n* = 2) analyzing single-component interventions have assessed this approach alone. Finally, point-of-sale and placement on shelf interventions’ impact on healthy purchasing and consumption are difficult to discern because both strategies are incorporated predominantly as part of multi-component interventions. Additional research examining the unique contribution of placement and point-of-sale interventions is needed.

Findings from studies evaluating increased stocking interventions demonstrated that on its own, this strategy does not significantly influence shoppers’ purchasing and consumption of healthier items [35,36,37]. While increased stocking did not yield significant effects on shoppers’ purchasing and consumption of healthy foods and beverages on its own, research demonstrates that this intervention strategy may be effective when combined with other intervention types in multi-component interventions. Thus, more research regarding the effectiveness of increased stocking both on its own and with other interventions is needed.

Two additional factors to consider when evaluating the success of interventions within grocery retail environments are their potential scalability and sustainability [91]. Across both single- and multi-component studies reviewed, results demonstrate that simple interventions with low information costs and minimal barriers to implementation are more effective than complex strategies at increasing healthy purchasing and consumption behaviors among shoppers. Simple intervention strategies are easily understandable and require little effort from customers, whereas more complex strategies can be difficult, and laborious, for customers to interpret. Additionally, simple intervention strategies not only benefit customers but also retailers, as they are both feasible to implement and able to be retained over time, potentially increasing intervention sustainability.

Approximately 90 percent of studies (*n* = 66) analyzed in this review were likely to have been conducted in retail environments that accepted SNAP benefits indicating a high degree of intervention scalability for increasing SNAP-eligible populations purchasing and consumption of healthier items.

## 5. Limitations and Future Directions

Although most studies reviewed were likely to have been conducted in SNAP-authorized retail settings, outcomes of SNAP participants can often not be identified separately from non-SNAP participants. To identify whether these intervention strategies are effective with SNAP populations specifically, future studies can attempt to directly measure interventions’ impact on SNAP shoppers’ purchasing and consumption rates.

Furthermore, of the 73 studies reviewed, 34 percent of studies (*n* = 25) were conducted without a control or comparison group (Table 2), indicating a need for more rigorous methodological designs in grocery retail environment research. Furthermore, 55 percent of studies reviewed were multi-component interventions (*n* = 40), and the complexity of these studies made it difficult to parse out the effects of individual intervention strategies on shoppers’ purchasing and consumption of healthy items.

Of the 33 single-component studies reviewed, only three of nine intervention types (e.g., nutrition scoring, increased stocking, nutritional messaging) were assessed in five or more studies. All other intervention types (e.g., non-nutritional messaging, food tasting and demos, nutrition education, endcaps and secondary placement, point-of-sale interventions, and placement on shelf) were evaluated in fewer than five single-component studies, highlighting a need for further evidence to determine their effectiveness. Thus, the frequent use of multi-component interventions, and the lack of studies evaluating single-component interventions, complicates the generalizability of findings within grocery retail environment literature.

Another factor not analyzed in this paper that likely contributes to the scalability and sustainability of healthy retail interventions includes intervention cost. Costly, time-consuming, interventions are unlikely to be maintained long-term and could prohibit broader adoption of these efforts. Future research evaluating the effect of intervention cost on SNAP shoppers’ purchasing and consumption outcomes is needed.

Currently, SNAP-authorized retailers must only meet minimum stocking standards. The USDA should test additional intervention strategies and grocery retailer eligibility requirements to evaluate whether they improve the health of grocery retail environments and whether they have an impact on grocery retailer participation. Additional requirements may be reasonable given substantive grocery retailer benefits from SNAP and would have the potential to positively impact the grocery retail environment for all, given widespread grocery retailer participation in SNAP.

## 6. Conclusions

This review identifies intervention strategies implemented within U.S. grocery retail stores that are effective in improving the healthfulness of shoppers’ food and beverage purchasing and consumption. Of the intervention strategies reviewed, nutritional scoring and nutritional messaging were the most rigorously tested and effective. Therefore, these strategies should be reviewed for policy implementation within SNAP-authorized grocery retailers. Additionally, research demonstrates that simple interventions yield the most successful results and minimize shopper burden. Other strategies, such as nutrition education and food tastings and demonstrations, were also effective, though more research is needed to corroborate existing findings. On its own, increased stocking did not significantly change participants’ purchasing or consumption behaviors; however, this strategy shows promise when implemented in conjunction with some other strategies in multi-component interventions. More research is needed to understand whether endcaps and secondary placement, point-of-sale, and placement on shelf are successful.

## Figures and Tables

**Table 1:** Description of Intervention Types Included in Review.

Intervention Type	Intervention Description
Multi-component interventions	Multi-component interventions signify a retailer’s simultaneous usage of at least two of the intervention types listed below.
Nutrition Scoring	Nutrition scoring interventions involved the development of a scale to represent the healthfulness of certain food and beverage items throughout the food retail environment.
Increased stocking	Increased stocking interventions specifically stocked a higher quantity of healthy food items.
Nutritional messaging	Nutritional messaging interventions utilized signage, flyers, or other promotional materials specifically noting the healthful benefits of certain.
Non-nutritional messaging	Non-nutritional messaging interventions tracked the effectiveness of non-traditional marketing strategies (e.g., scarcity labeling and floor labeling to guide shoppers to healthier store sections).
Food tasting and demonstrations	Food tasting and demonstrations represent interventions in which participants were given healthy food to sample and/or were shown how to prepare a recipe with healthier ingredients sold in-store.
Nutrition education	Nutrition education interventions involved “any set of learning experiences designed to facilitate the voluntary adoption of eating and other nutrition-related behaviors conducive to health and well-being” (Washington State Department of Social and Health Services, 2022).
Endcaps and secondary placement	Endcaps and secondary placement interventions promoted healthier items in a display placed at the end of an aisle or in other locations in addition to their primary placement throughout the store (e.g., in a newly installed refrigeration unit).
Point-of-sale interventions	Point-of-sale interventions promoted healthy food items in retail checkout lines or counters (healthy checkout interventions). Point-of-sale interventions differ from point-of-purchase (POP) interventions, which often signify all in-store interventions and are not limited to the checkout vicinity.
Placement on shelf	Placement on shelf interventions promoted healthy food items by manipulating the location of healthy food items on store shelves, often moving healthier items to eye level or placing them on more prominent shelves within the store.

**Table 2:** Study Design Features of Included Articles by Intervention Type.

Study Design Feature	Total (*n* = 73)	Multi-Component Interventions (*n* = 40)	Nutrition Scoring (*n* = 11)	Increased Stocking (*n* = 6)	Nutritional Messaging (*n* = 5)	Non-Nutritional Messaging (*n* = 4)	Food Tasting and Demos (*n* = 2)	Nutrition Education (*n* = 2)	Endcaps and Secondary Placement (*n* = 2)	Point-of-Sale Interventions (*n* = 1)
***Study Design***										
*Experimental*	23 (32%)	16 (40%)	1 (9%)	--	2 (40%)	2 (50%)	1 (50%)	--	1 (50%)	--
*Quasi-experimental*	25 (34%)	11 (28%)	5 (45%)	3 (50%)	2 (40%)	2 (50%)	--	--	1 (50%)	1 (100%)
*Pre-experimental*	25 (34%)	13 (33%)	5 (45%)	3 (50%)	1 (20%)	--	1 (50%)	2 (100%)	--	--
Objective data *	43 (59%)	17 (43%)	10 (91%)	4 (66%)	4 (80%)	4 (100%)	1 (50%)	--	2 (100%)	1 (100%)
Subjective data **	30 (41%)	23 (58%)	1 (9%)	2 (33%)	1 (20%)	--	1 (50%)	2 (100%)	--	--
***Population***										
*SNAP enrollees*	24 (33%)	14 (35%)	--	4 (66%)	1 (20%)	2 (50%)	1 (50%)	--	1 (50%)	1 (100%)
*Low-income community*	20 (27%)	15 (38%)	2 (18%)	1 (16%)	1 (20%	1 (25%)	--	--	--	--
*Bodega or corner store*	7 (10%)	5 (13%)	--	--	--	--	--	--	1 (50%)	--
*Major supermarket*	15 (21%)	3 (8%)	8 (73%)	--	1 (20%)	1 (25%)	--	2 (100%)	--	--

* Objective data refers to methodological tracking of sales and/or purchases of healthy items using store sales receipts or other store records. ** Subjective data refers to study participants’ self-reported sales, purchasing, and/or intake/consumption of healthy items.

**Table 3:** Intervention Types in Multi-Component Interventions.

Rigor	Study Name	Increased Stocking	Endcaps & Secondary Placement	Nutrition Education	Food Tasting and Demos	Nutritional Messaging	Non-Nutritional Messaging	Placement on Shelf	Nutrition Scoring	Point-of-Sale
**EXP + OBJ**	Milliron, 2012			X					X	
	Gittelsohn et al., 2017	X		X	X	X *				
	Williams et al., 2021	X	X			X				
	Thorndike et al., 2017	X					X	X		
	Wensel et al., 2019		X	X			X			
	Foster et al., 2014		X		X	X		X		
	Banerjee & Nayak, 2018			X	X					
**Total (EXP + OBJ)**		**3**	**3**	**4**	**3**	**3**	**2**	**2**	**1**	**0**
**EXP + SELF**	Ayala et al., 2022		X	X	X					X
	Martinez-Donate et al., 2015	X			X	X				X
	Lent et al., 2014	X		X		X	X			
	Trude et al., 2018	X		X	X	X				
	Bird Jernigan et al., 2019	X	X				X			
	Shin et al., 2015			X	X			X		
	Trude et al., 2019	X		X						
	Ayala et al., 2013		X		X	X				
	Gittelsohn et al., 2013			X	X					
**Total (EXP + SELF)**		**5**	**3**	**6**	**5**	**4**	**2**	**1**	**0**	**2**
**QUAS + OBJ**	Surkan et al., 2016	X		X	X				X	
	Kannan et al., 2020			X		X		X		
	Gustafson, Ng et al., 2019		X				X			
	Jilcott Pitts et al., 2018	X	X					X		
	Holmes et al., 2012		X		X			X		
**Total (QUAS + OBJ)**		**2**	**3**	**2**	**2**	**1**	**1**	**3**	**1**	**0**
**QUAS + SELF**	Gittelsohn et al., 2010			X			X		X	
	Mackenzie et al., 2019		X						X	X
	Schultz & Litchfield, 2016				X	X				
	Steeves et al., 2015	X		X		X				
	Ortega et al., 2016	X	X				X			
	Albert et al., 2017	X	X				X			
**Total (QUAS + SELF)**		**3**	**3**	**2**	**1**	**2**	**3**	**0**	**2**	**1**
**PRE + OBJ**	Woodward-Lopez et al., 2018		X		X		X	X		
	Boys et al., 2021	X	X							
	Lawman et al., 2015	X		X		X		X		
	Gudzune et al., 2015	X					X			
	Paluta et al., 2019	X				X				
**Total (PRE + OBJ)**		**4**	**2**	**1**	**1**	**2**	**2**	**2**	**0**	**0**
**PRE + SELF**	Sutton et al., 2019				X	X			X	
	Beckelman et al., 2020				X	X		X		
	Rushakoff et al., 2017		X		X	X				
	Gustafson, McGladrey et al., 2019				X		X	X		
	Paek et al., 2014	X		X			X			
	Liu et al., 2017		X				X	X		
	Davis et al., 2016		X		X			X		
	Dannefer et al., 2012	X	X							
**Total (PRE + SELF)**		**2**	**4**	**1**	**5**	**3**	**3**	**4**	**1**	**0**
**Grand Total**		**19**	**18**	**16**	**17**	**15**	**13**	**12**	**5**	**3**

Note: The rigor column groups articles according to the study design and outcomes measured. Study design was coded as experimental, quasi-experimental, or pre-experimental. Outcome measures were coded as either using objective purchasing data or subjective purchasing data. An X indicates the intervention type utilized in a study. * Limited information about messaging type; category assumed.

## Appendix A

**Table A1:** Sales, Purchasing, and Intake/Consumption Results of Multi-Component Intervention Studies.

Study Name	Sales Outcomes	Purchasing Outcomes	Intake/Consumption Outcomes
Milliron, 2012	(+) Fresh fruit (+/null) Fresh vegetables		
Gittelsohn et al., 2017	(+/null) Healthy beverages, dairy, fruits and vegetables (fresh and dried), grains, nuts and seeds, protein, salty snacks		
Williams et al., 2021	(+/−) Fruits and vegetables (fresh, canned, or frozen) (+/null) Salads		
Thorndike et al., 2017	(+) Fruits and vegetables	(−) Fruits and vegetables	
Wensel et al., 2019	(+/−/null) Healthy juice, dairy, fruits and vegetables (fresh), grains, infant foods and formula, protein		
Foster et al., 2014	(+/null) Dairy (−) Cereal (+/−) Healthy frozen meals		
Ayala et al., 2022		(+) Fruit (fresh)	(+/null) Fruit (fresh)
Martinez-Donate et al., 2015		(+/null) Fruits and vegetables	
Lent et al., 2014		(null) Healthy grains, beverages, snacks, protein	
Trude et al., 2018		(+/null) Healthy dairy, grains, snacks, and fruits and vegetables (fresh or canned)	
Bird Jernigan et al., 2019			(null) Fruits and vegetables, protein, snacks
Shin et al., 2015		(+/−/null) Healthy snacks, grains, nuts and seeds, and fruits and vegetables	
Trude et al., 2019		(null) Fruits and vegetables (fresh or canned)	(+/null) Fruits and vegetables (fresh or canned)
Gittelsohn et al., 2013		(+/null) Healthy grains	(+/null) Healthy grains
Surkan et al., 2016	(+) Healthy grains, beverages, fruits and vegetables, snacks, and dairy		
Kannan et al., 2020	(null) Healthy beverages, legumes, vegetables (fresh), and grains	(+) Healthy beverages, legumes, vegetables (fresh), and grains	
Gustafson, Ng et al., 2019	(null) Fruits and vegetables		(null) Fruits and vegetables
Jilcott Pitts et al., 2018	(null) Fruits and vegetables (fresh, canned, or frozen)		(null) Fruits and vegetables (fresh, canned, or frozen)
Holmes et al., 2012	(+) Fruits and vegetables (fresh), Healthy grains, nuts and seeds, (−/null) Dairy and vegetables		
Gittelsohn et al., 2010		(+) Healthy grains, dairy, fruits and vegetables, and beverages	(+/null) Healthy grains, dairy, fruits and vegetables, and beverages
Mackenzie et al., 2019		(+) Fruits and vegetables (fresh or frozen)	
Schultz & Litchfield, 2016			(+) Fruit and vegetables, healthy grains, and protein
Steeves et al., 2015		(+) Healthy grains, dairy, snacks, and fruits and vegetables	
Ortega et al., 2016		(null) Fruits and vegetables	(null) Fruits and vegetables
Albert et al., 2017		(null) Fruits and vegetables (fresh, canned, and frozen)	(null) Fruits and vegetables (fresh, canned, and frozen)
Woodward-Lopez et al., 2018	(+/−/null) Fruits and vegetables	(+) Fruits and vegetables	
Boys et al., 2021	(+/null) Dairy products (+) Whole grains (+) nuts, high protein items (+) Fruits (+) Vegetables (−) Fruit Juice		
Lawman et al., 2015	(null) Healthy grains, protein, snacks, and beverages	(null) Healthy grains, protein, snacks, and beverages	
Gudzune et al., 2015	(+) Fruits and vegetables (fresh)		
Paluta et al., 2019	(+) Healthy proteins, grains, and fruits and vegetables (fresh, canned, or frozen) *		
Sutton et al., 2019		(null) Fruits and vegetables	
Beckelman et al., 2020		(+/null) Fruit (fresh) and healthy beverages	
Rushakoff et al., 2017		(+) Healthy beverages, dairy, grains, nuts and seeds, and fruits ad vegetables (fresh, canned, or frozen)	(+) Vegetables (fresh)
Gustafson, McGladrey et al., 2019			(+) Fruits (+) Vegetables
Paek et al., 2014		(+) Healthy beverages, dairy, fruits and vegetables (fresh), grains, snacks, nuts and seeds, legumes	(+/null) Healthy grains (+) Nuts and seeds, legumes (null) Beverages, dairy, fruits and vegetables (fresh)
Liu et al., 2017		(+) Fruits and vegetables	(+) Fruits and vegetables
Davis et al., 2016		(+) Healthy grains, frozen meals, and dairy	
Ayala et al., 2013		(+/null) Fruits and vegetables (fresh, canned, and frozen)	
Banerjee & Nayak, 2018	(+, null) Fruits and vegetables (fresh), grains		
Dannefer et al., 2012		(+) Healthy dairy, snacks, grains, and fruits and vegetables (fresh and canned)	

Note: Study outcomes are categorized as related to either sales, purchasing, or intake/consumption. Sales outcomes denote the use of store-level data tracking customer sales data. Purchasing outcomes are results obtained from the perspective of the customer often through post-shopping surveys. Intake/consumption outcomes are related to studies reporting healthy item consumption rates. Outcomes are coded as either positive (+), negative (−), or null based on the intervention’s effect on healthy item sales, purchasing, or intake/consumption. Positive results represent an increase in healthy item sales, purchasing, or consumption, negative results represent a decrease in healthy item sales, purchasing, or consumption and null results represent no change in healthy item sales, purchasing, or consumption. * Data were missing for one evaluation period. During this time, decreases in items sold were observed.
